# Proteome analysis revealed the essential functions of protein phosphatase PP2A in the induction of Th9 cells

**DOI:** 10.1038/s41598-020-67845-2

**Published:** 2020-07-03

**Authors:** Suyasha Roy, Renu Goel, Suruchi Aggarwal, Shailendra Asthana, Amit Kumar Yadav, Amit Awasthi

**Affiliations:** 1 Immuno-Biology Laboratory, Translational Health Science and Technology Institute (THSTI), 3rd Milestone Gurgaon-Faridabad Expressway, Faridabad, Haryana 121 001 India; 2 Drug Discovery Research Centre, Translational Health Science and Technology Institute, Faridabad, Haryana India

**Keywords:** CD4-positive T cells, Skin cancer

## Abstract

Proteomic analysis identifies post-translational functions of proteins, which remains obscure in transcriptomics. Given the important functions of Th9 cells in anti-tumor immunity, we performed proteome analysis of Th9 cells to understand the involvement of proteins that might be crucial for the anti-tumor functions of Th9 cells. Here we performed a comprehensive proteomic analysis of murine Th0 and Th9 cells, and identified proteins that are enriched in Th9 cells. Pathway analysis identified an abundance of phosphoproteins in the proteome of Th9 cells as compared to Th0 cells. Among upregulated phosphoproteins, Ppp2ca (catalytic subunit of protein phosphatase, PP2A) was found to be highly enriched in Th9 cells. Although the role of PP2A has been shown to regulate the differentiation and functions of Th1, Th2, Th17 and Tregs, its role in the differentiation and functions of Th9 cells is not identified yet. Here we found that PP2A is required for the induction of Th9 cells, as PP2A inhibition leads to the suppression of IL-9 and expression of key transcription factors of Th9 cells. PP2A inhibition abrogates Th9 cell-mediated anti-tumor immune response in B16-OVA melanoma tumor model. Thus, we report that PP2A is essential for the differentiation and anti-tumor functions of Th9 cells.

## Introduction

Cytokines such as TGF-β1 and IL-4 together skew naïve CD4^+^ T cells to IL-9-producing T helper (Th) cells, named as “Th9” cells^1,2^. Th9 cells play a critical role in promoting anti-tumor immunity, allergic inflammation and autoimmune diseases^3,4^. So far, different transcription factors have been reported to play a crucial role in the development of Th9 cells^5–11^, however, these factors have also been reported in other effector T cell subsets and are not lineage specific-transcription factors for Th9 cells. Understanding the generation of Th9 cells will allow modulating effector functions of these cells during the pathophysiology of asthma and anti-tumor immune responses. Thus, identification of biomarkers for Th9 cells is crucial for developing diagnostics and therapy for promoting the clinical outcomes for patients with Th9-mediated diseases.

For deciphering the complete identity of a cell, quantitative proteomics is an important tool, as there is a discordance between transcriptome and proteome due to post-translational modifications and differential rates of protein synthesis. The mRNA level of a gene is poorly correlated with the protein levels in different conditions due to various above mentioned reasons^12^. Therefore, despite of several transcriptome analysis, global proteome profiling has gained significant importance for mapping protein signatures at cellular level to unravel their precise functional characterization. There have been significant advancements in the development of proteome for different immune cells. Although the mass spectrometry studies on Th cells are rare, still few studies have reported the proteome analysis of different effector T cell subsets ^13–17^.

Rautajoki et al. has demonstrated the differential protein expression patterns in human Th1 and Th2 cells^15^. They identified 14 differentially expressed proteins in human Th1 and Th2 cells among which N-terminal acetylation of cyclophilin A was significantly higher in Th1 as compared to Th2 cells^15^. Riaz et al. identified the proteome of gut-derived human Th1 and Th1/Th17 clones from Crohn’s disease patients and identified 334 differentially expressed proteins with majority of cytotoxic proteins being overrepresented in Th1 clones than in mixed phenotype Th1/Th17 clones. The Th1 clones showing cytotoxic features were CD28^+^ NKG2D^−^ CD4^+^ Th1 cells and constituted only a subgroup when tested from seven different Crohn’s disease patients^16^. There are studies showing quantitative proteomic analysis of human regulatory T cells^13^ and during human Th17 cell polarization^12^. Recently published study has revealed the differentially expressed proteins in multiple sclerosis patients through quantitative proteomics of CD4^+^ and CD8^+^ T cells^18^. However, at present no such studies have investigated the proteomic analysis of Th9 cells.

Here we identified, through proteomic analysis, protein phosphatase PP2A as one of the differentially upregulated protein in murine Th9 cells. We show, here, that PP2A is essential for Th9 cell differentiation and Th9-cell mediated anti-tumor immunity.

## Methods

### Mice

C57BL/6 (#00064) wild type (WT) and OT-II TCR (#004194) mice were procured from Jackson laboratory, housed and maintained in pathogen-free small animal facility at Translational Health Science and Technology Institute, Faridabad, India. All the mice used for experiments were 6–12 weeks old and both age and sex matched. All animal experiments were performed in accordance to the THSTI Animal Ethical guidelines and the experimental protocols were approved by institutional (THSTI ) animal ethics committee (IAEC).

### T cell differentiation

6–12 weeks old WT mice were euthanized, spleen and lymph nodes (LN) were collected aseptically. Single cell suspension was prepared, then stained with the antibodies- anti-mouse CD4 PerCP (Biolegend; 100538), anti-mouse CD62L APC (Biolegend; 104412) and anti-mouse CD25 PE (Biolegend; 101904). Cells were sorted on BD FACS Aria with > 98% purity. Sorted purified naïve (CD4^+^CD62L^+^) T cells were activated with plate bound anti-CD3 (2.0 μg/ml; 145-2C11; Bioxcell) and anti-CD28 (2.0 μg/ml; 37.51; Bioxcell) in 96 well flat bottom plates and in vitro differentiated into Th0 (without cytokines) and Th9 cells in the presence of TGF-β1 (2.0 ng/ml; 240-B, R&D), IL-4 (20 ng/ml; 214-14, Peprotech) and anti-IFN-γ (XMG1.2, BioXcell) for 3 days with or without Okadaic acid (OA; Tocris; 1136) or LB-100 (Selleckchem; S7537).

For Intracellular cytokine staining, cells were re-stimulated with PMA (phorbol 12-myristate13-acetate; 50 ng/ml; Sigma-Aldrich), ionomycin (1.0 μg/ml; Sigma-Aldrich) and monensin (#554724; GolgiStop, BD Biosciences) for 6 h. Cells were stained with anti-mouse CD4 (Biolegend; RM4-5) and anti-mouse CD8α (Biolegend; 53-6.7) antibodies followed by live/dead staining. Cells were stained with anti-mouse IL-17A (BioLegend; TC11-18H10), anti-mouse IL-9 (RM9A4, BioLegend) or anti-mouse IFN-γ (BioLegend; XMG1.2). Cells were acquired on FACSVerse (BD biosciences) and the results were analyzed with FlowJo software (Tree star).

### SDS-PAGE and In-gel digestion

30 µg proteins from each of Th0 and Th9 cells were resolved by 10% SDS-PAGE followed by commassie blue staining. 21 gel pieces were excised for each of the Th0 and Th9 conditions and in-gel digestion was carried out as described previously^19,20^. The excised bands were destained with 40 mM ammonium bicarbonate (ABC) in 40% acetonitrile (ACN). The gel bands were processed further for reduction using 5.0 mM dithiothreitol (DTT) (60 °C for 45 min) and alkylation using 20 mM iodoacetamide (IAA) (room temperature for 10 min in dark)^19,20^. The gel sections were dehydrated with 100% ACN followed by trypsin digestion for 10–12 h at 37 °C (Gold mass-spectrometry trypsin; Promega)^19,20^. The peptides were extracted from the gel pieces with 0.4% formic acid twice, once in 50% ACN solution and once in 100% ACN. The peptides were then vacuum-dried and stored at − 80 °C for LC–MS/MS analysis^19,20^.

### LC–MS/MS analysis

All fractions were subjected to 5,600 Triple-TOF mass spectrometer which is directly linked to reverse-phase high-pressure liquid chromatography Ekspert-nanoLC 415 system (Eksigent). The trap column (200 μm × 0.5 mm) and the analytical column (75 μm × 15 cm) were both from Eksigent, packed with 3 μm ChromXP C-18 (120 Å), and were used for reverse phase elution by Ekspert-nanoLC 415 system^20^. Mobile phase A was 0.1% formic acid in water and 0.1% formic acid in ACN was used as mobile phase B. The elution of fractions were performed from the analytical column at a flow rate of 250 ml/min using an initial gradient elution of 10% B from 0 to 5 min, transitioned to 40% over 120 min, ramping up to 90% B for 5 min, holding 90% B for 10 min, followed by re-equilibration of 5% B at 10 min with a total run time of 150 min^20^.

Peptides were injected into the mass spectrometer using 10 μm Silica Tip electrospray Pico Tip emitter (New Objective Cat. No. FS360-20-10-N-5-C7-CT), and the ion source was operated with the following parameters: ISVF = 1950; GS1 = 20; CUR = 12. The data dependent acquisition experiments was set to obtain a high resolution TOF–MS scan over a mass range 100–1,250 *m/z*, followed by MS/MS scans of 20 ion candidates per cycle with activated rolling collision energy, operating the instrument in high sensitivity mode using the Analyst TF 1.7 where each 1 s MS survey scan was followed by 3 MS/MS scans of 3 s. The selection criteria for the parent ions included the intensity, where ions have to be greater than 150 cps, with a charge state between + 2 and + 5, mass tolerance of 50 mDa and on a dynamic exclusion list were present^20^.

Mass spectra (MS) and tandem mass spectra (MS/MS) were recorded in positive-ion and high-sensitivity mode with a resolution of ~ 35,000 full-width half-maximum. Before running samples in the mass spectrometer, spectra calibration was done after acquisition of every sample using dynamic LC–MS and MS/MS acquisitions of 100 fmol β-galactosidase. Once an ion had been fragmented by MS/MS, its isotopes were excluded from further MS/MS fragmentation for 12 s. The ion accumulation time was set to 250 ms (MS) and to 70 ms (MS/MS). The collected raw files spectra were stored in .wiff format.

### Data analysis for mass spectrometry data

Raw mass spectrometry files were searched in MaxQuant (version 1.6.7.0) software against a UniProt mouse protein database (June, 2018). Carboamidomethylation of cysteine was used as fixed modification while methionine oxidation and N-terminal acetylation were used as variable modification^21^. MaxQuant search algorithm uses separate mass tolerances for each peptide, however, the initial maximum precursor mass tolerances were set to 20 ppm in the first search and 4.5 ppm in the main search. The fragment mass tolerance was set to 20 ppm^21^. Further, following parameters were used: instrument type 5,600, minimum peptide length was set as 7 and maximum peptide mass was 4,600 Da, centroid half width 35 ppm, label free quantification (LFQ) was checked with LFQ minimum ratio count 2, maximum allowed missed cleavages 1 and trypsin was specified as protease.

The output of this search was a .txt file which consists of protein name, accession, number of common and unique peptides, molecular weight of the proteins, sequence coverage by mass-spectrometry identification, MS/MS counts and iBAQ (intensity-based absolute quantification). The unique and razor peptides were used for the quantification of proteins. iBAQ intensities were calculated using MaxQuant software^12^. We filtered the data to keep only highly reliable stringent proteins quantitation values by using following parameters which includes: (1) threshold of 1% accepted FDR proteins; (2) ≥ 5 razor and unique peptides; and (3) absolute iBAQ > 20 intensity, was considered for analysis. Fold change (FC) was calculated by using the formula: [Th9(iBAQ)/Th0(iBAQ)]. Log10 FC values were then calculated and threshold was set as 10 FC (which was ± 1 at log_10_ scale). Proteins identified only in Th9 cells were considered highly expressed and assigned a log_10_ FC value of + 3 and for proteins highly expressed in Th0 cells were assigned a log_10_ FC value of -3.

### Gene ontology (GO) and pathway analysis

The top upregulated proteins at log_10_ FC =  + 1 were subjected to GO and pathway analysis. GO analysis was done using PANTHER (https://pantherdb.org/)^22^ and pathway analysis was done using STRING 11 database server (https://string-db.org/)^23^.

### Protein–protein interaction network construction

For the identification of functional protein enrichment and associations, the network of key genes was built by using the STRING 11 database^23^. The corresponding protein–protein interaction network was constructed when we selected the interactions pertaining to mouse and showed minimum interactions with a confidence score > 0.9. Differentially expressed proteins were used to create two separate networks—one for upregulated proteins while the other for downregulated proteins. The networks were manually analyzed to find out signaling related hub proteins that may be central to Th9 cell differentiation.

### Real-time PCR

RNA isolation from T cells was performed using the RNAeasy Mini Kit (Qiagen; #74104) and reverse transcribed into cDNA using the iScript cDNA synthesis kit (Biorad; #1708891). RT-PCR was done using the KAPA SYBR^®^ FAST qPCR Master Mix (2×) Universal Kit (KK4600) on Fast 7500 Dx real-time PCR system (Applied Biosystems) according to manufacturer’s protocol. The relative gene expression of each target gene was calculated after normalization to the internal control (*β*-actin) as described previously^24^. The following SYBR primers were used for the analysis: *β*-actin (forward 5′-GATGTATGAAGGCTTTGGTC-3′; reverse 5′-TGTGCACTTTTATTGGTCTC-3′), *Il9* (forward 5′-CTGATGATTGTACCACACGTGC-3′; reverse 5′-GCCTTTGCATCTCTGTCTTCTGG-3′), *Spi1* (forward 5′-CATGAGGTGAAATGTGAGAG-3′); reverse (5′-AGTTGGTTGAAATGGATCAC-3′), *Irf4* (forward 5′-ACGCTGCCCTCTTCAAGGCTT-3′; reverse 5′-TGGCTCCTCTCGACCAATTCC-3′), *Hif1α* (forward 5′-CGATGACACAGAAACTGAAG-3′; reverse 5′-GAAGGTAAAGGAGACATTGC-3′), *Batf* (forward 5′-AAAATGACAAGTCAACCCTG-3′; reverse 5′-TTAGAAAACTATCCACCCCC-3′), *Gata3* (forward 5′-TATTAACAGACCCCTGACTATG-3′; reverse 5′-CACCTTTTTGCACTTTTTCG-3′), *Irf1* (forward 5′-TCTGTATAACCTACAGGTGTC-3′; reverse 5′- CAGACTGTTCAAAGAGCTTC -3′) and *Foxo1* (forward 5′-CCGGAGTTTAACCAGTCCAA-3′; 5′-TGCTCATAAAGTCGGTGCTG-3′).

### In-vitro T cell proliferation assay

Naive CD4^+^ T cells were stained with 5.0 μM CFSE (carboxyfluorescein diacetate succinimidyl ester; Life Technologies), and differentiated into Th9 in the presence or absence of increasing doses of LB-100 (0, 1, 2, 5) μM for 3 days. Cell proliferation was assessed by flow cytometry at the end of culture^25^.

### Knockdown by siRNA transfection

Naive CD4^+^ T cells were transfected with silencer select predesigned 25 nM siRNA specific for mouse PP2A (#AM16708, Ambion, Life Technologies) or silencer negative control scramble siRNA (#AM4611, Ambion, Life Technologies) with transfection reagent (#MIR 2155, Trans-IT-TKO Transfection Reagent, Mirus) according to the manufacturer’s instruction^8^ and were then differentiated into Th0 and Th9 respectively for further analysis.

### B16-OVA melanoma model

2 × 10^5^ B16-OVA cells were subcutaneously injected into flank region of WT mice for melanoma development. 2 × 10^6^ OVA-specific OT-II-Th9 cells ± LB-100 were transferred intravenously into B16-OVA-tumor bearing mice at day 7. Mice were then randomized into following groups: Group I: mice injected with B16-OVA cells only (B16-OVA); Group II: mice injected with B16-OVA and adoptively transferred OT-II-Th9 cells (B16-OVA + Th9); and Group III: mice injected with B16-OVA and adoptively transferred OT-II-Th9 cells differentiated in the presence of LB-100 (B16-OVA + Th9 + LB-100). Tumor growth was monitored and tumor volume was measured using vernier caliper. Tumor volume was calculated as: Volume (mm^3^) = L × W^2^/2, where L is the length and W is the width of the tumor (in mm). Mice were euthanized when the tumor volume exceeded 2000 mm^3^ or there was severe skin necrosis defined as the end-point of the study^4,8^. At the end point, spleen and tumor draining lymph nodes and TILs were isolated^26^. Cells were re-stimulated ex vivo with PMA/ionomycin followed by intracellular cytokine staining in CD4^+^ and CD8^+^ T cell populations^4,8^.

### Statistical analysis

One-way ANOVA for comparison of means between more than two groups and two-way ANOVA test for comparison among multiple groups with two variables was used with Tukey’s multiple comparison’s test for all statistical analysis using GraphPad Prism 7.0. *P* value < 0.05 was considered statistical significant for all the experiments. All the data are represented as mean ± SEM.

## Results

### LC–MS/MS based analysis of differentially expressed proteins in Th9 cells

**Figure 1:**
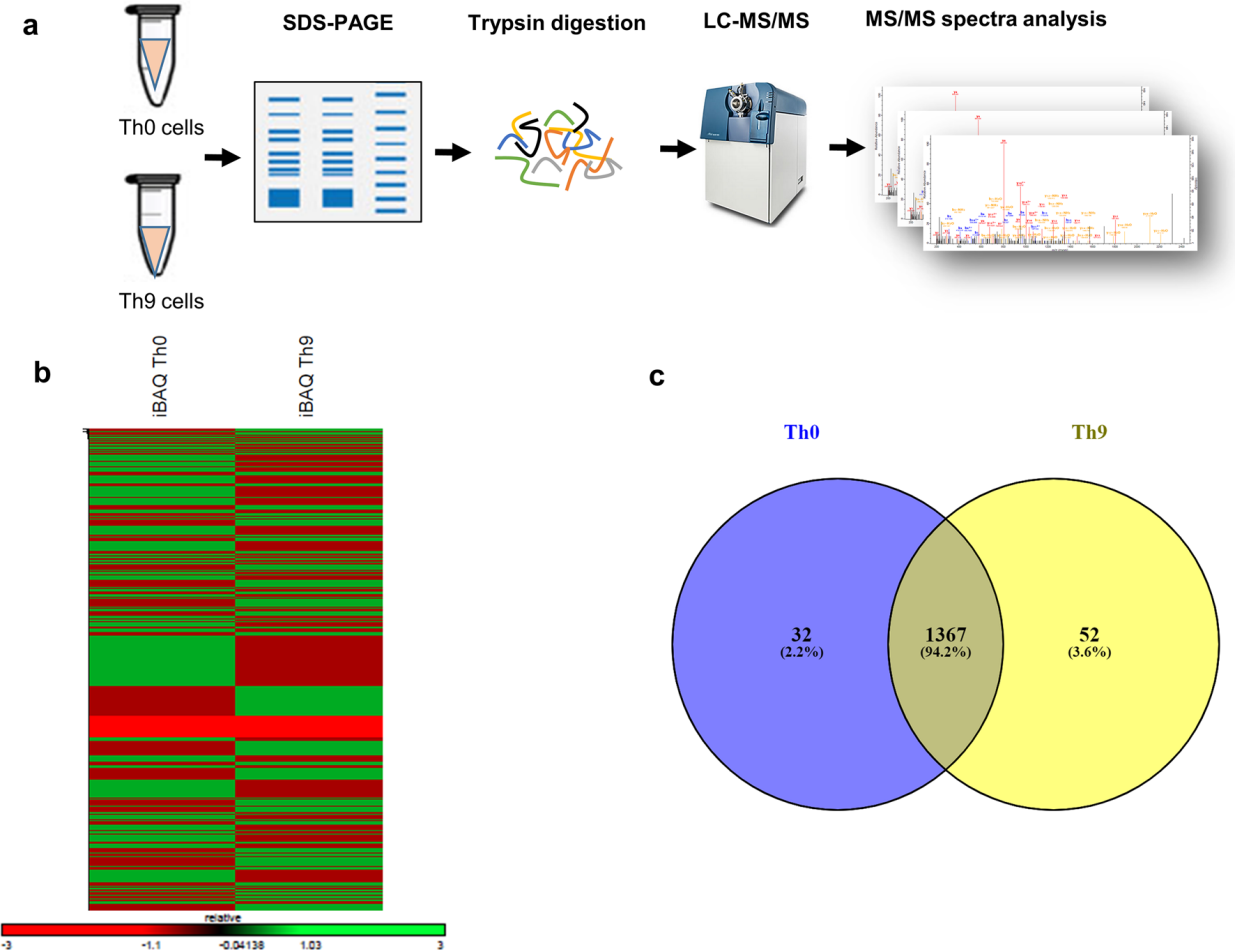
LC–MS/MS based analysis of differentially expressed proteins in Th9 cells. (**a**–**c**) Naïve CD4^+^ T cells from WT mice were in vitro differentiated into Th0 and Th9 conditions. Cells were lysed for SDS-PAGE followed by in-gel digestion and LC–MS/MS analysis. (**a**) Schematic representation of the proteomic workflow employed for the study. (**b**) Heatmap for the Z-score from iBAQ intensities for proteins in Th0 and Th9 cells. Z-score was calculated from raw absolute intensities as shown in the heatmap. (**c**) Venn diagram showing comparison of proteins between Th0 and Th9 cells at ≥ 5 peptide and > 20 iBAQ intensity cut-offs.

Transcriptomics data identified essential factors that are required for differentiation and functions of Th9 cells. However, transcriptomics analysis of Th9 cells failed to capture the proteins that are modulated by post-translational modifications such as phosphorylation, ubiquitination and acetylation. To understand the proteome of Th9 cells, we performed proteome analysis, using in-gel digestion and liquid chromatography-mass spectrometry (LC–MS), of Th9 cells and compared it to the proteome of Th0 cells. This experimental design, as represented in Fig. 1a, allowed us to generate the map of differentially expressed proteins in Th9 cells.

It has been shown that IFN-γ interferes with Th9 differentiation^27^, so we polarized Th9 cells in the presence of TGF-β1 and IL-4 together with the increasing concentrations of anti-IFN-γ antibody, however we did not find any difference in the IL-9 levels upon IFN-γ neutralization (Supplementary Fig. 1). Therefore, in accordance with the previous studies, we followed the usual protocol of Th9 cell differentiation using TGF-β1 and IL-4 for all the experiments conducted in this study^2,5,9^. LC–MS/MS analysis showed a differential pattern of protein abundance in Th0 and Th9 cells, as shown by iBAQ intensity **(**Fig. 1b). The number of proteins at various peptide cut-offs was used to evaluate the proteome yield (Supplementary Table S1). To rule out any false positives in proteome analysis, we used the stringency criteria of ≥ 5 peptides for the identification and quantification of proteins in Th0 and Th9 cells for further analysis. The most stringent criteria i.e. the cut-off of ≥ 5 peptides identified protein counts of 1,195 and 1,163 in Th0 and Th9 cells respectively (Supplementary Table S1). In addition, we used another filter, cut-off of > 20 iBAQ intensity for increasing the stringency in order to generate high quality data. This led to the identification of a total of 1,451 proteins, out of which 1,367 proteins were common in Th0 and Th9 cells while 52 and 32 proteins were uniquely identified in Th9 and Th0 cells respectively (Fig. 1c).

### Gene ontology-based classification of proteins in Th9 cells

**Figure 2:**
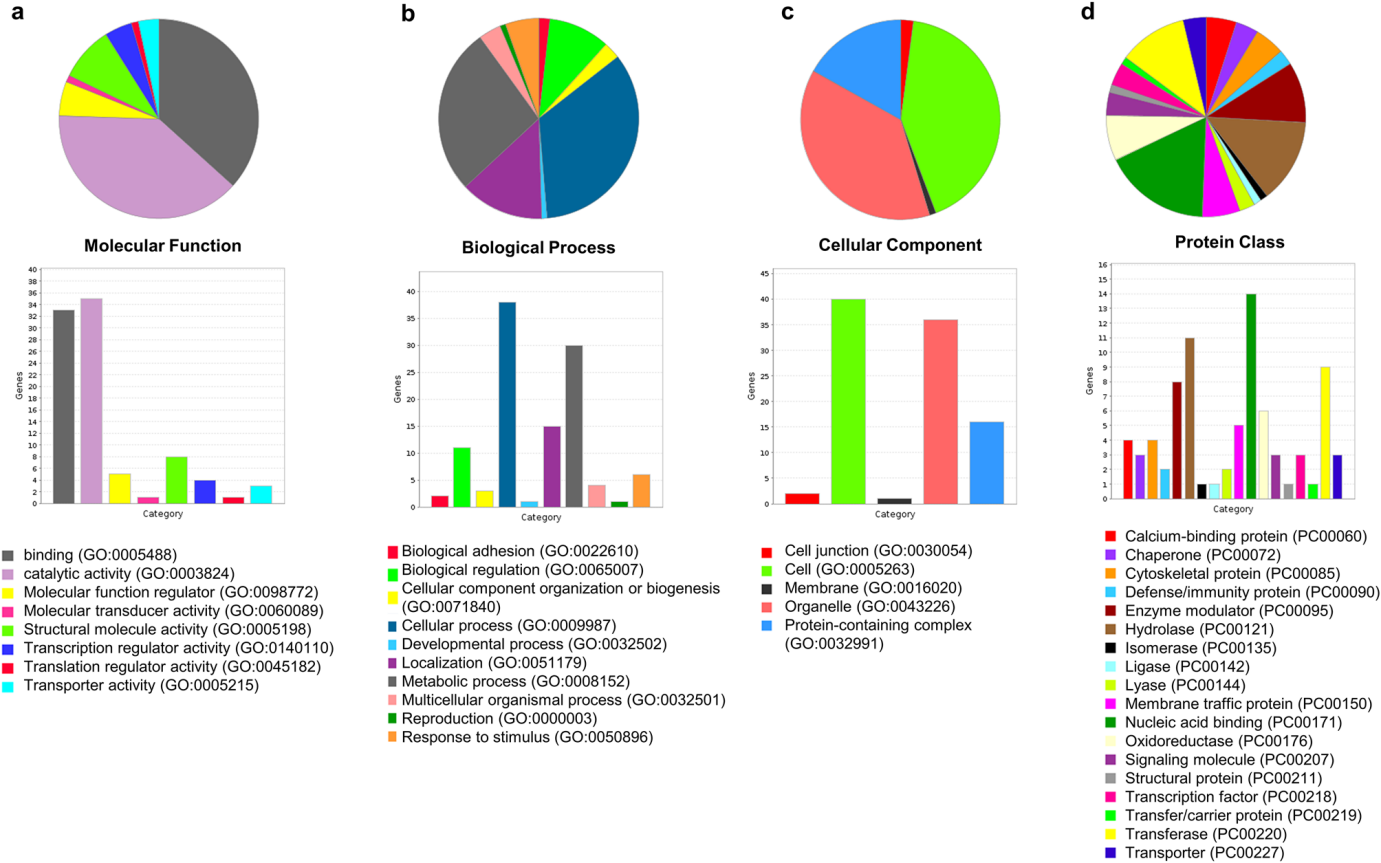
Gene ontology-based classification of proteins in Th9 cells. Pie-chart showing functional categorization of top upregulated proteins in Th9 cells using PANTHER. (**a**) Molecular function, (**b**) biological process, (**c**) cellular component, (**d**) protein class.

Next, we categorized the proteins into gene ontology based classification using GO analysis. We increased stringency criteria by using another filter of fold change of differential expression at FC = 10 (log_10_ FC ± 1). This resulted in the identification of 118 upregulated proteins (Supplementary Table S2) and 81 downregulated proteins (Supplementary Table S3) in Th9 cells. The top 118 upregulated proteins were then subjected to GO analysis for gaining deeper biological insights and were categorized on the basis of molecular functions, biological process, cellular components and protein class (Fig. 2a–d).

### Proteome interaction network of Th9 cells

**Figure 3:**
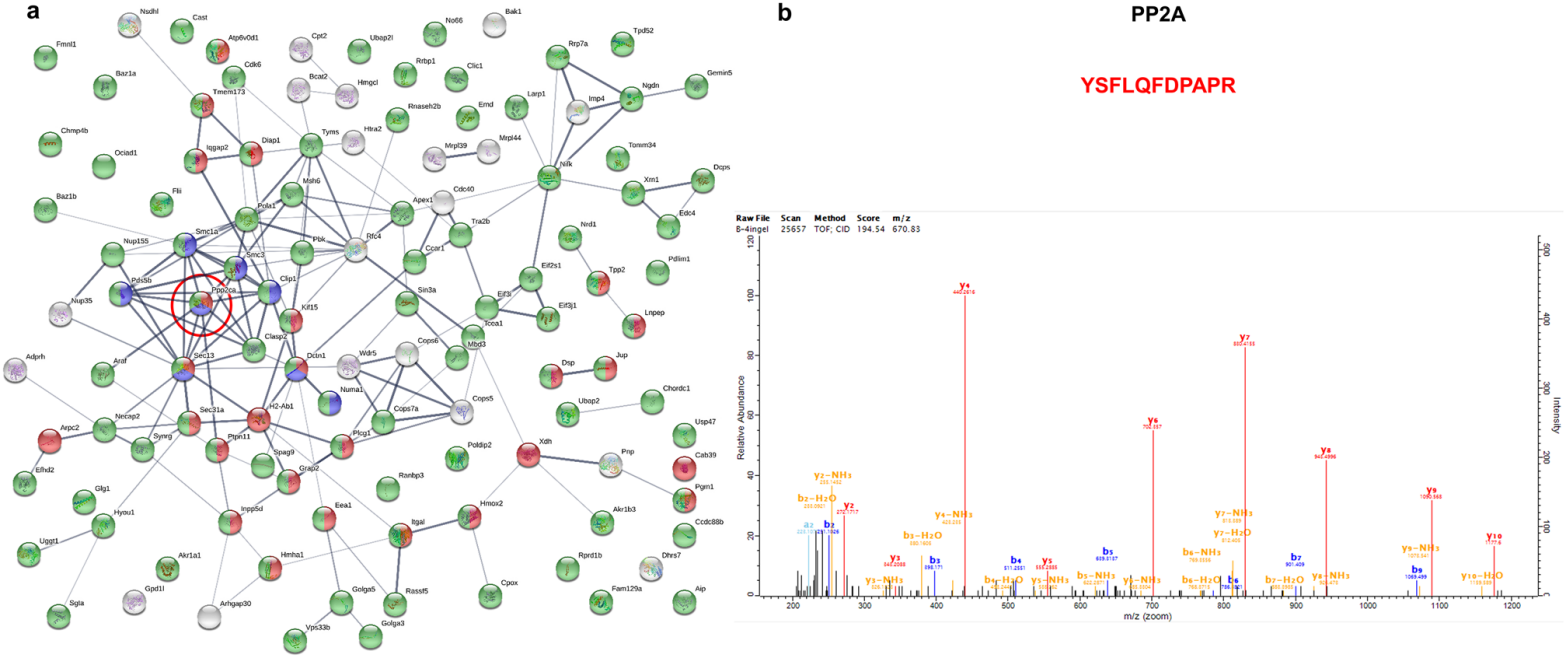
Proteome interaction network of Th9 cells. (**a**) STRING based protein–protein interaction network of upregulated proteins in Th9 cells at > 20 iBAQ intensity cut-off (red-immune system; blue-mitotic pro-metaphase; green-phosphoproteins). PP2A is encircled in red. (**b**) Representative MS/MS spectra of PP2A peptide identified in Th9 cells.

To identify the protein interactome of Th9 cells, protein–protein interaction networks for upregulated and downregulated proteins were created using STRING database. We also fetched the pathways for the upregulated and downregulated proteins in Th9 cells using Reactome Pathways in STRING analysis**.** We tabulated the pathways involving upregulated (Supplementary Table S4) and downregulated (Supplementary Table S5) proteins in Th9 cells. Among 181 upregulated proteins, we found striking abundance of phosphoproteins in Th9 cells (Fig. 3a). Since phosphoproteins cannot be studied at gene expression level, therefore we specifically focused on them. Among the phosphoproteins, Ppp2ca (catalytic subunit of PP2A; red circle) was identified as an abundant phosphoprotein which showed to be involved in 25 different pathways (Supplementary Table S4). We generated a network of upregulated phosphoproteins involved in immune system and mitotic pro-metaphase (as reference) and found a dense interaction-network of Ppp2ca with other phosphoproteins clearly reflecting its dominant presence in Th9 cells. We manually checked the spectral quality and a representative MS/MS spectra of a PP2A peptide is shown (Fig. 3b). We decided to further investigate the role and functions of PP2A in the differentiation and functions of Th9 cells. In addition, we also generated protein-interaction network of downregulated proteins in Th9 cells (Supplementary Fig. S2).

### PP2A is essential for IL-9 induction in Th9 cells

**Figure 4:**
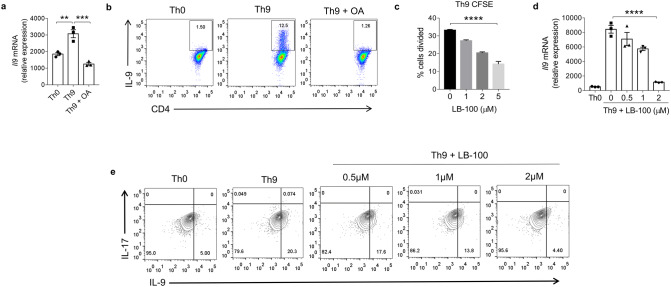
PP2A is essential for IL-9 induction in Th9 cells. (**a**, **b**) Naïve CD4^+^ T cells from WT mice were in vitro differentiated to Th0 and Th9 with or without 200 nM okadaic acid (OA) followed by (**a**) RT-PCR analysis of *Il9* mRNA expression and (**b**) flow cytometric analysis of intracellular IL-9 production. (**c**) CFSE labelling for in vitro proliferation of Th9 cells in the presence of LB-100 dose titration. Percentage of cells divided was plotted against concentration of LB-100. (**d**,**e**) Naïve CD4^+^ T cells were polarized to Th0 and Th9 in the absence or presence of increasing concentrations of LB-100 (0, 0.5, 1, 2 μM). (**d**) *Il9* mRNA expression was measured by RT-PCR and (**e**) FACS analysis of intracellular staining for IL-9 and IL-17. Data are representative of mean ± SEM from three independent experiments (n = 3). **P < 0.0021, ***P < 0.0002, ****P < 0.0001; one-way ANOVA followed by Tukey’s multiple comparison test (**a**,**c**,**d**).

Although PP2A has been shown to play a crucial role in the differentiation and functions of Th2, Th17 and Tregs^25,26,28^, its role in the differentiation of Th9 cells has not been identified. As indicated by our proteome analysis of Th9 cells, we further tested the role of PP2A in the generation of Th9 cells. To decipher the role of PP2A in Th9 cell differentiation, we pharmacologically inhibited PP2A using 200 nM okadaic acid (OA) in Th9 cells and found a decrease in *Il9* expression by qPCR as well as reduction in the intracellular IL-9 production in Th9 cells (Fig. 4a,b). Since okadaic acid is a non-specific PP2A inhibitor, so we further used LB-100, which is a specific PP2A inhibitor, during the differentiation of Th9 cells. Notably, increasing doses of LB-100 suppressed the proliferation of Th9 cells as measured by the percentage of dividing cells (Fig. 4c and Supplementary Fig. S3a). However, there was no detrimental effect of LB-100 on the cell survival as shown by the percentage of live cell cells population in Th9 cell condition, which remains the same with different doses of LB-100 (Supplementary Fig. S3b). Consistently, qPCR analysis showed that inhibition in the expression of *Il9* with increasing concentrations of LB-100 in Th9 cells, suggesting a dose dependent inhibition of IL-9 induction in Th9 cells with LB-100 (Fig. 4d). Similarly, intracellular expression of IL-9 was also substantially decreased at 2.0 μM of LB-100 in Th9 cells (Fig. 4e). Taken together, these data indicates that PP2A is essential for IL-9 induction in Th9 cells, as pharmacological inhibition of PP2A led to a dramatic reduction of IL-9 in Th9 cells.

### PP2A inhibition impairs Th9 cell differentiation

**Figure 5:**
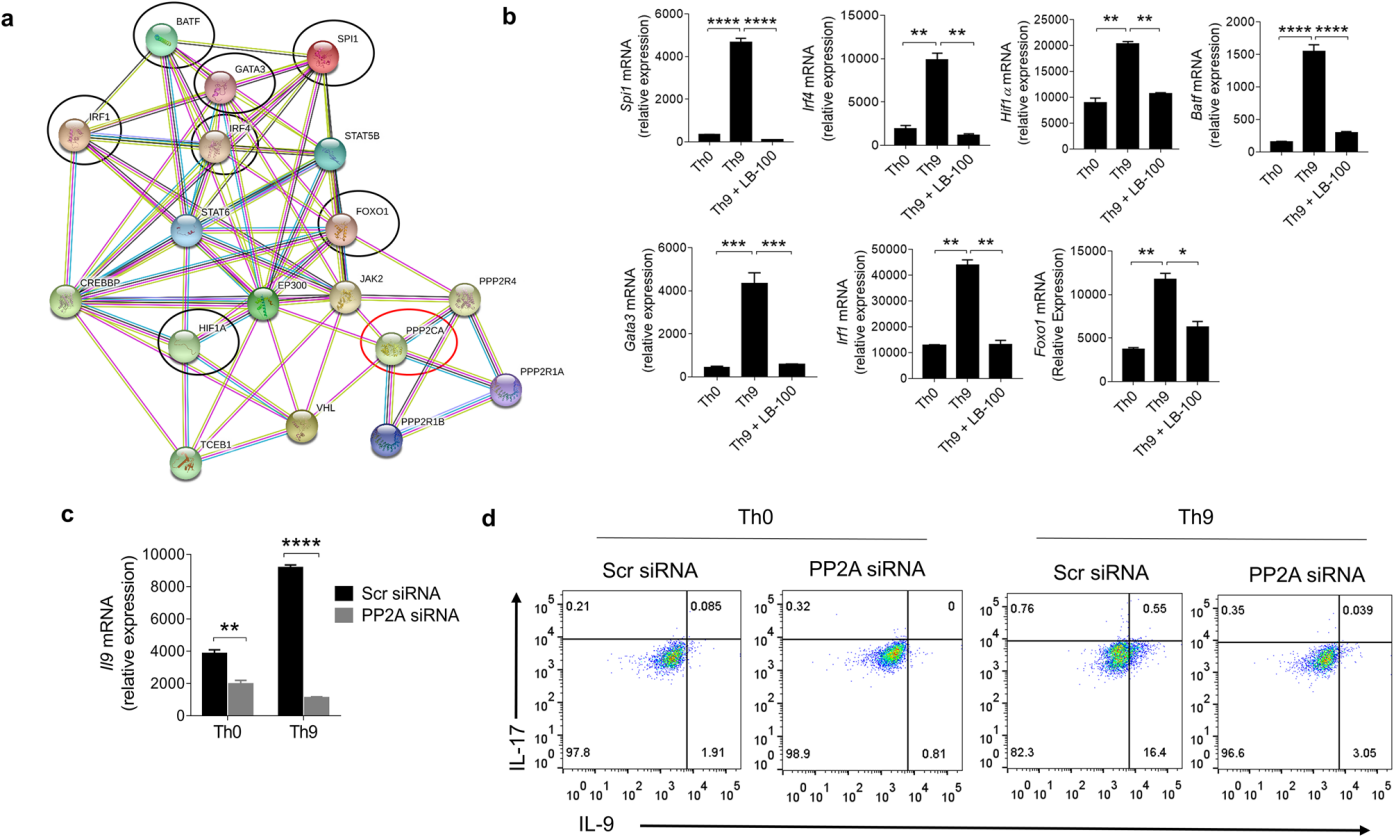
PP2A inhibition impairs Th9 cell differentiation. (**a**) STRING based predictive PP2A interaction network. (**b**) Naïve CD4^+^ T cells were in vitro differentiated to Th0 and Th9 with or without 2.0 μM LB-100 followed by RT-PCR analysis of *Spi1, Irf4, Hif1α, Batf, Gata3, Irf1* and *Foxo1* expression. (**c**,**d**) Naïve CD4^+^ T cells were transfected with scramble (Scr) siRNA or PP2A siRNA and in vitro differentiated to Th0 and Th9 respectively. (**c**) *Il9* expression was measured by RT-PCR, and (**d**) intracellular staining for IL-9 and IL-17. Data are representative of mean ± SEM from two to three independent experiments (n = 3). *P < 0.0332, **P < 0.0021, ***P < 0.0002, ****P < 0.0001; one-way ANOVA followed by Tukey’s multiple comparison test (**b**); two-way ANOVA followed by Tukey’s multiple comparison test (**c**).

To further evaluate the role of PP2A in the transcriptional programming of Th9 cells, we generated, using STRING database, a prediction-based interactome to identify the putative interactions of PP2A with known transcription factors of Th9 cells such as PU.1 (*Spi1*)^10^, IRF4^11^, HIF1α^9^, BATF^7^, GATA3^6^, IRF1^8^ and Foxo1^5^. PP2A interactome suggests a potential direct and indirect association of PP2A with the key transcription factors of Th9 cells such as PU.1 (*Spi1*), IRF4, HIF1α, BATF, GATA3, IRF1 and Foxo1, all of these transcription factors are found to be prevalent in Th9 cells (Fig. 5a). To validate this observation, we, in vitro, tested whether the inhibition of PP2A also blocked the expression of these transcription factors that are essential in Th9 cell differentiation and functions. Interestingly, our qPCR data clearly demonstrated that PP2A inhibition with LB-100 suppressed the expression of *Spi1*,* Irf4*,* Hif1α*,* Batf*,* Gata3*,* Irf1* and *Foxo1* in Th9 cells (Fig. 5b), suggesting that PP2A is one of the essential regulatory node which is required for the expression of key transcription factors, a requisite for Th9 cell differentiation.

In addition, to establish the functional role of PP2A in Th9 cells, we genetically knockdown PP2A using siRNA to elucidate its functionality apart from using chemical inhibitors (okadaic acid and LB-100). Knockdown of PP2A in Th9 cells showed a significant reduction in IL-9 induction at both mRNA and protein levels as compared to control siRNA (Fig. 5c, d). PP2A knockdown also attenuates IL-9 induction in the control Th0 group, however the silencing effect of PP2A on IL-9 induction was more pronounced in Th9 cells as compared to Th0 (Fig. 5c, d). All together, these data suggests that inhibition of PP2A impairs Th9 cell differentiation.

### PP2A is required for anti-tumor functions of Th9 cells in vivo

**Figure 6:**
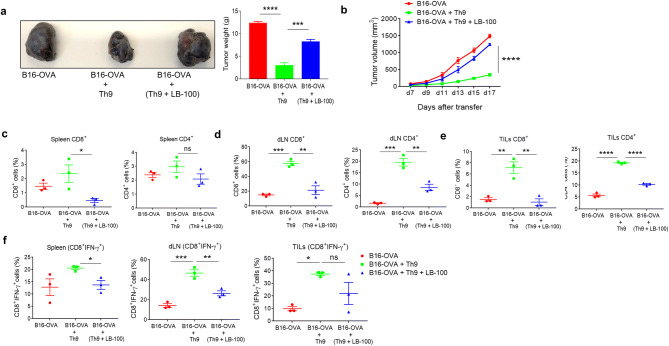
PP2A is required for anti-tumor functions of Th9 cells in vivo*.* (**a**–**f**) Naïve CD4^+^ T cells from OT-II TCR transgenic mice were differentiated to Th9 with or without 2 μM LB-100 for 3 days. Cells were then adoptively transferred into B16-OVA-tumor bearing WT mice (n = 3 mice per group). (**a**) At the end point of study, mice were sacrificed and tumor was aseptically removed and tumor size and weight was recorded. (**b**) Tumor growth curve with mean tumor volume over time. (**c**–**f**) TILs were isolated from the tumor while spleen and tumor draining lymph nodes (dLN) were harvested and single cell suspensions were made followed by (**c**–**e**) percentage of total CD8^+^ and CD4^+^ cell populations in (**c**) spleen, (**d**) dLN and (**e**) TILs; (**f**) Percentage of CD8^+^ IFN-γ^+^ T cell populations in spleen, dLN and TILs. Data are representative of mean ± SEM from three independent experiments (n = 3). *P < 0.0332, **P < 0.0021, ***P < 0.0002, ****P < 0.0001, ns-not significant; one-way ANOVA followed by Tukey’s multiple comparison test (**a**–**f**).

To further establish the functional and therapeutic role of PP2A in Th9 cells in vivo, we used B16-OVA melanoma tumor model, as Th9 cells have shown to possess potent anti-tumor property^4,8,29^. To understand whether inhibition of PP2A abrogates the anti-tumor functions of Th9 cells, naïve CD4^+^ T cells from OT-II TCR transgenic mice differentiated into Th9 cells in the presence or absence of LB-100. These cells were then adoptively transferred into a B16-OVA tumor bearing mice on day 7, and the tumor progression was monitored. It was found that tumor weight and volume was higher in the group of mice which received LB-100-treated OT-II Th9 cells as compared to the group of mice which received OT-II-Th9 cells alone (Fig. 6a, b), suggesting that the inhibition of PP2A abrogates the anti-tumor functions of Th9 cells.

To further understand the cellular mechanism of abrogation of anti-tumor function of Th9 cells with LB-100 treatment, we examined the frequencies of T cells in spleen and tumor draining lymph nodes (dLN). In spleen, the frequency of CD8^+^ T cells was found to be increased with the transferred OT-II-Th9 cells while the frequency of CD8^+^ T cells was decreased in LB-100-treated OT-II Th9 cells group (Fig. 6c). There was no significant difference in the frequency of CD4^+^ T cells among all 3 groups in the spleen (Fig. 6c). Interestingly, in dLN, the frequency of both CD8^+^ and CD4^+^ T cells were significantly decreased in (OT-II-Th9 + LB-100) group as compared to OT-II-Th9 group (Fig. 6d) indicating that Th9 cells treated with LB-100 suppressed the effector populations, especially CD8^+^ T cells, that are essential for regressing tumor growth. Similarly, there was a reduction in the frequency of both CD8^+^ and CD4^+^ T cells in tumor-infiltrating lymphocytes (TILs) in the (OT-II-Th9 + LB-100) group as compared to Th9 group (Fig. 6e). These data indicates that PP2A is required for the anti-tumor functions of Th9 cells.

The anti-tumor functions of Th9 cells is mediated by IFN-γ and IL-21 while IL-9 could also directly kill tumor cells^8,29,30^, thus we tested whether PP2A mediated inhibition of Th9 cells abrogates its anti-tumor functions via suppressing IFN-γ production. OT-II-Th9 group treated with LB-100 showed a decrease in the IFN-γ expression in CD8^+^ T cells in spleen, dLN and TILs as compared to OT-II-Th9 group respectively (Fig. 6f) (Supplementary Fig. S4a–c). There was a reduction in the frequency of CD4^+^ IFN-γ^+^ cells in TILs, however, there was no significant difference in the frequency of CD4^+^ IFN-γ^+^ cells in the spleen and dLN (Supplementary Fig. S5). Taken together, these data suggests that CD8^+^ T cells are the major effector population producing IFN-γ in the tumor microenvironment. Adoptive transfer of Th9 cells induces anti-tumor immune response by CD8^+^ T cells, which gets significantly abrogated upon PP2A inhibition by LB-100.

## Discussion

There are reports for proteomic analysis of different T cell subsets, however, the proteomic analysis of Th9 cells remain unexplored. In the present study, we have characterized the proteome of murine Th9 cells through profiling of differentially expressed proteins comparative to Th0 cells. We identified a total of 1,451 proteins in both Th9 and Th0 cells, out of which 1,367 proteins were common while 52 proteins in Th9 and 32 proteins in Th0 cells were uniquely identified. Gene-ontology based analysis grouped proteins into four functional categories: molecular functions, biological process, cellular components and protein class. Molecular function classified proteins into 8 groups with the predominance of proteins having binding and catalytic activity. Biological process classified proteins into 10 groups with the predominance of proteins involved in cellular and metabolic process. Cellular components categorized proteins into 5 groups with majority of proteins localized within cell and organelles. Proteins were grouped into 17 protein classes, among which majority of the proteins were nucleic acid binding, hydrolases, transferases and enzyme modulators.

Further analysis of Th9 cell proteome showed 118 upregulated and 81 downregulated proteins after manual filtering. Pathway analysis of upregulated proteins revealed a dominance of phosphoproteins in the proteome of Th9 cells. Interestingly, pathway analysis of 81 downregulated proteins also revealed an abundance of phosphoproteins suggesting the fact that phosphoproteins is critically involved in the development of Th9 cells.

Protein–protein interaction network further identified Ppp2ca (catalytic subunit of PP2A) as the most connected phosphoprotein in Th9 cells among 22 phosphoproteins and has been shown to be involved in T cell differentiation. Ppp2ca was shown to be upregulated in 25 different pathways, while Ptpn11 in 19, Plcg1 in 14 and Sec13 in 13 different pathways, among other highly expressed proteins in Th9 cells. Among top downregulated pathways manually picked, phosphoproteins showed strong protein–protein interactions with proteins which were either nucleotide-binding or involved in ubiquitination or both.

Protein Phosphatase-2A (PP2A) is a highly conserved heterotrimeric serine-threonine phosphatase which consists of three distinct subunits—the scaffold A subunit (PP2AA), the regulatory B subunit (PP2AB) and the catalytic C subunit (PP2AC), which assembled together into a trimolecular complex^31,32^. The PP2A holocomplex takes part in the regulation of key cellular processes, such as cellular metabolism, cell cycle, apoptosis, migration, proliferation and differentiation, and is involved in the development of cancer^33^, SLE^34^ and neurodegenerative diseases^35^. PP2A modulates several signaling cascades, including the PI3K-AKT-mTOR ^36,37^, MAPK^38,39^, and NF-κB pathways.

PP2A has been well described in the differentiation of Th1, Th2, Th17 and Tregs ^25,26,28^, however the link between PP2A and Th9 cells is not known. The role of PP2A is well characterized in generation and functions of Tregs, as PP2A is essential for IL-2 receptor mediated signaling in Tregs^40^. Moreover, PP2A in Tregs is also essential for STAT5 mediated functions in Tregs^41^. Given the shared factor that are required for the generation of Tregs (TGF-β1) and Th9 (TGF-β1 + IL-4), PP2A seems to regulate the pathways that are essential for the generation and functions of Th9 cells. It is known that both IL-2-IL-2R signaling and STAT5 are essential component in the generation and functions of Th9 cells^42,43^. IL-2 or STAT5-deficiency leads to the inhibition of generation of Th9 cells^42,43^. Our data suggests that, similar to Tregs, PP2A is required for the induction of Th9 cells. It could be possible that inhibition of PP2A in Th9 cells might affect IL-2 and STAT5 mediated signaling which is crucial for the functions of Th9 cells.

Our data identified the role of PP2A in Th9 cell differentiation, as pharmacological inhibition of PP2A with okadaic acid or LB-100 led to impairement in the differentiation of Th9 cells. We observed a dose-dependent response of LB-100 in which higher doses (5.0 μM) reduced the proliferation of Th9 cells while an optimal dose of 2 μM was effective in inhibiting PP2A mediated IL-9 induction with lesser reduction in Th9 cell proliferation. We showed a probable interaction of PP2A with the critical transcription factors of Th9 cells such as Spi1^10^, IRF4^11^, HIF1α^9^, BATF^7^, GATA3^6^, IRF1^8^ and Foxo1^5^ through PP2A prediction-based interactome*.* Our data indicated that inhibition of PP2A downregulated the expression of *Spi1*,* Irf4*,* Hif1α*,* Batf Gata3*,* Irf1* and *Foxo1* in Th9 cells. This suggests that PP2A interaction with PU.1, IRF4, HIF1α, BATF, GATA3, IRF1 and Foxo1 could be crucial for Th9 cell functions. Our data further demonstrated that siRNA mediated knockdown of PP2A led to diminished IL-9 induction emphasizing the pivotal role of PP2A in Th9 cell differentiation.

Finally, we validated the in vivo role of PP2A in Th9 cell-mediated anti-tumor immune response. We found that inhibition of PP2A with LB-100 abrogated the anti-tumor response of Th9 cells when these cells were adoptively transferred to B16-OVA tumor bearing mice. This was due to the decreased infiltration of CD8^+^ T cells secreting IFN-γ population in the tumor. Therefore, we report here for the first time that PP2A is essential for the differentiation and anti-tumor functions of Th9 cells.

## Supplementary information

Supplementary file1

## Data Availability

The mass spectrometry proteomics data have been deposited to the ProteomeXchange Consortium via the PRIDE partner repository with the dataset identifier PXD019506.
