# Supplementary material for: Proteome analysis revealed the essential functions of protein phosphatase PP2A in the induction of Th9 cells
Source: Sci Rep. 2020 Jul 3;10:10992. doi: 10.1038/s41598-020-67845-2 (PMC7335106; doi:10.1038/s41598-020-67845-2)
Supplement: Supplementary file 1 — Supplementary file1 [file 41598_2020_67845_MOESM1_ESM.pdf]

Proteome analysis revealed the essential functions of protein phosphatase PP2A in the induction of Th9 cells

Suyasha Roy, Renu Goel, Suruchi Aggarwal, Shailendra Asthana, Amit Kumar Yadav, Amit Awasthi

Supplementary Figure S1

a

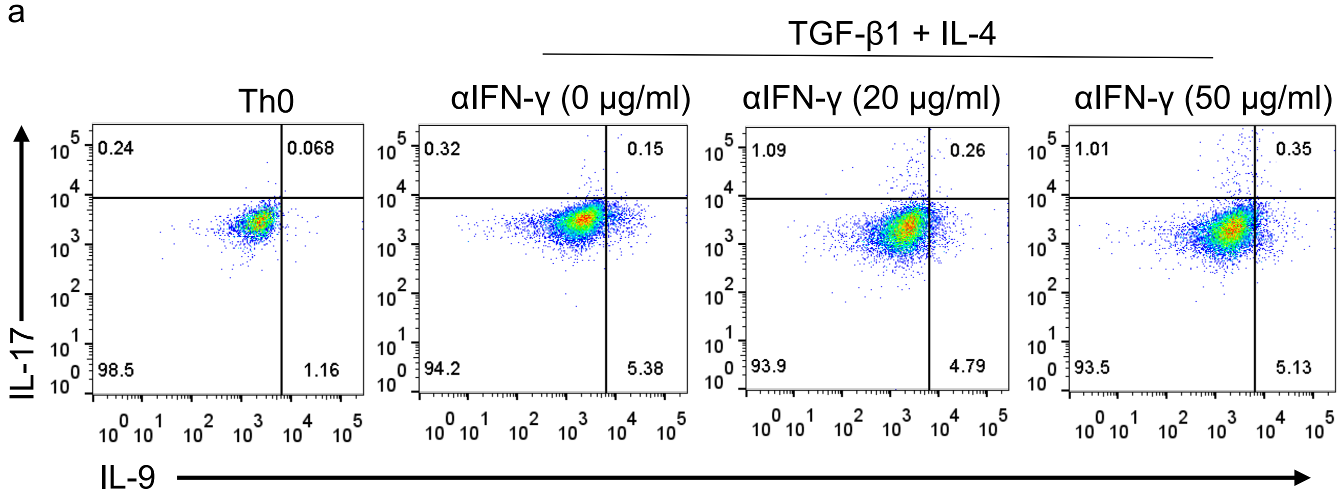

Supplementary Figure S2

a

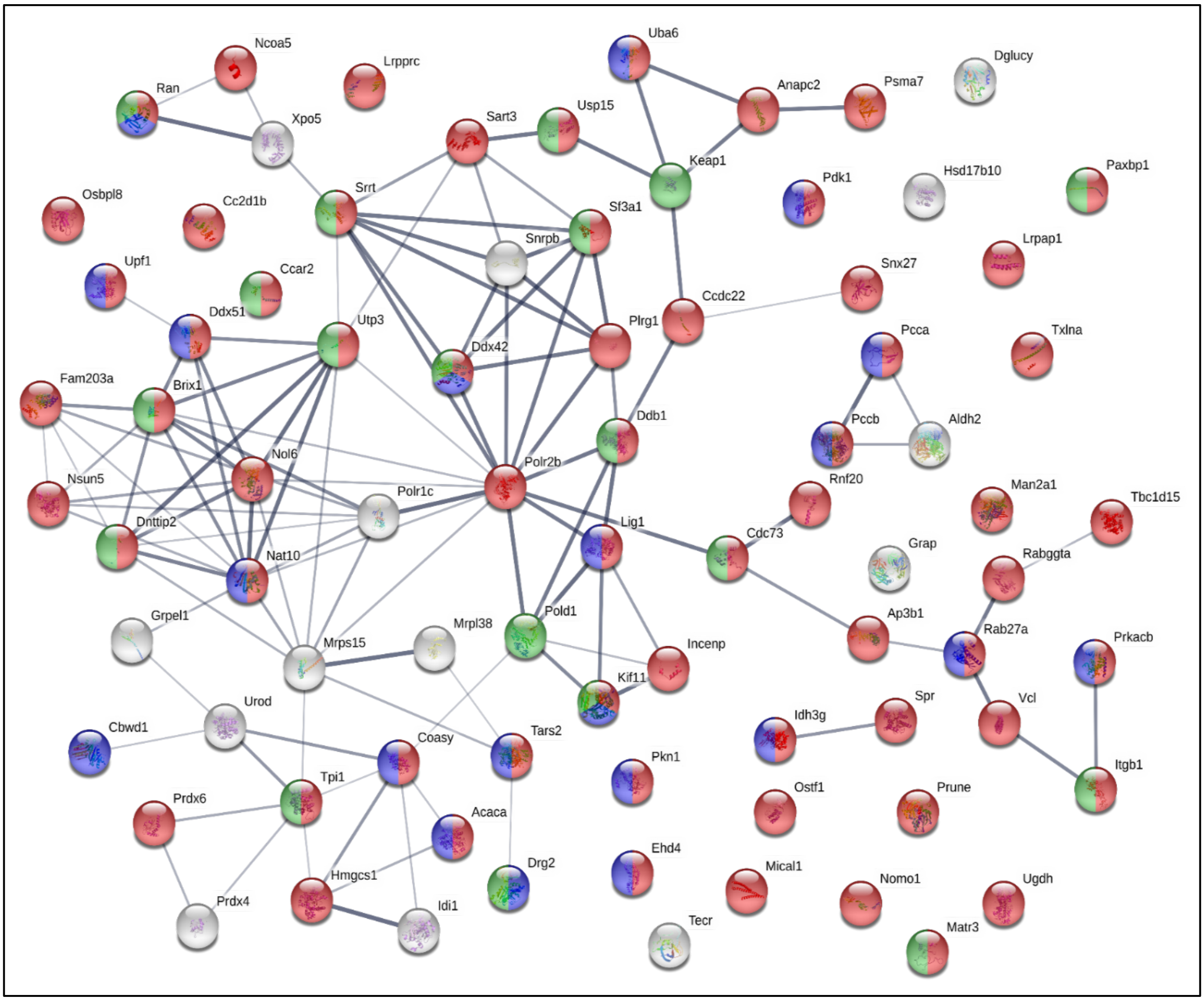

Supplementary Figure S3

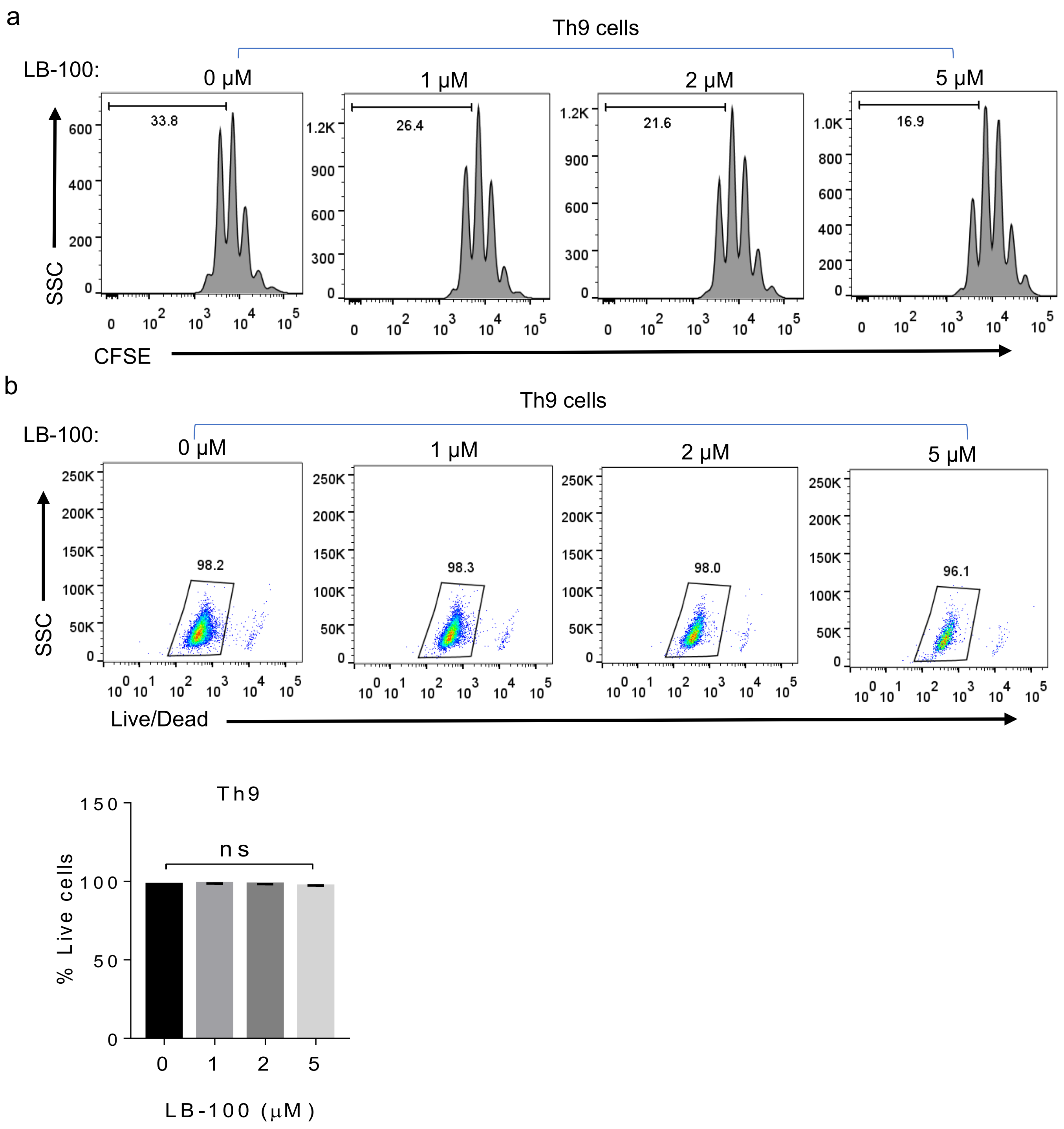

Supplementary Figure S4

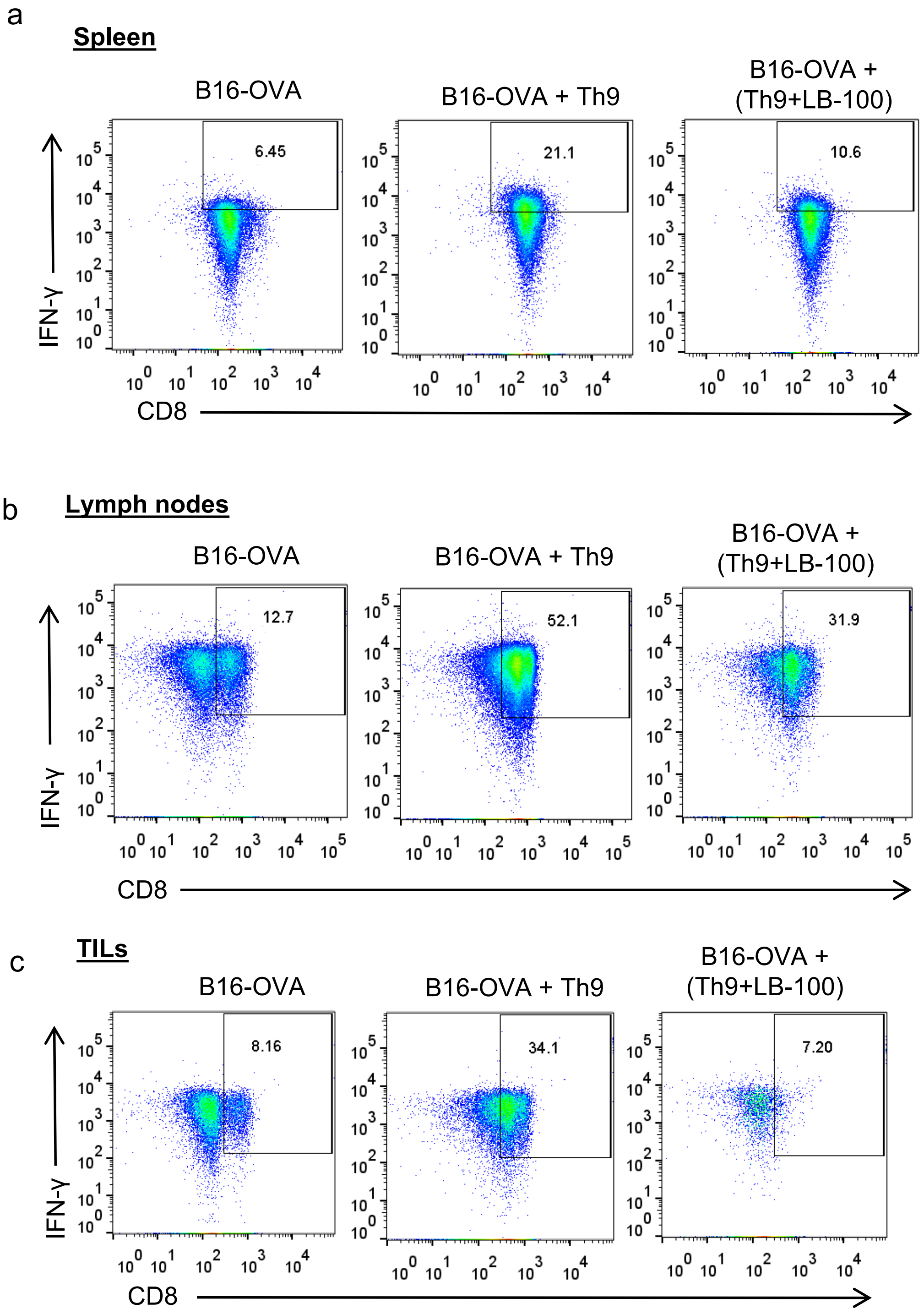

Supplementary Figure S5

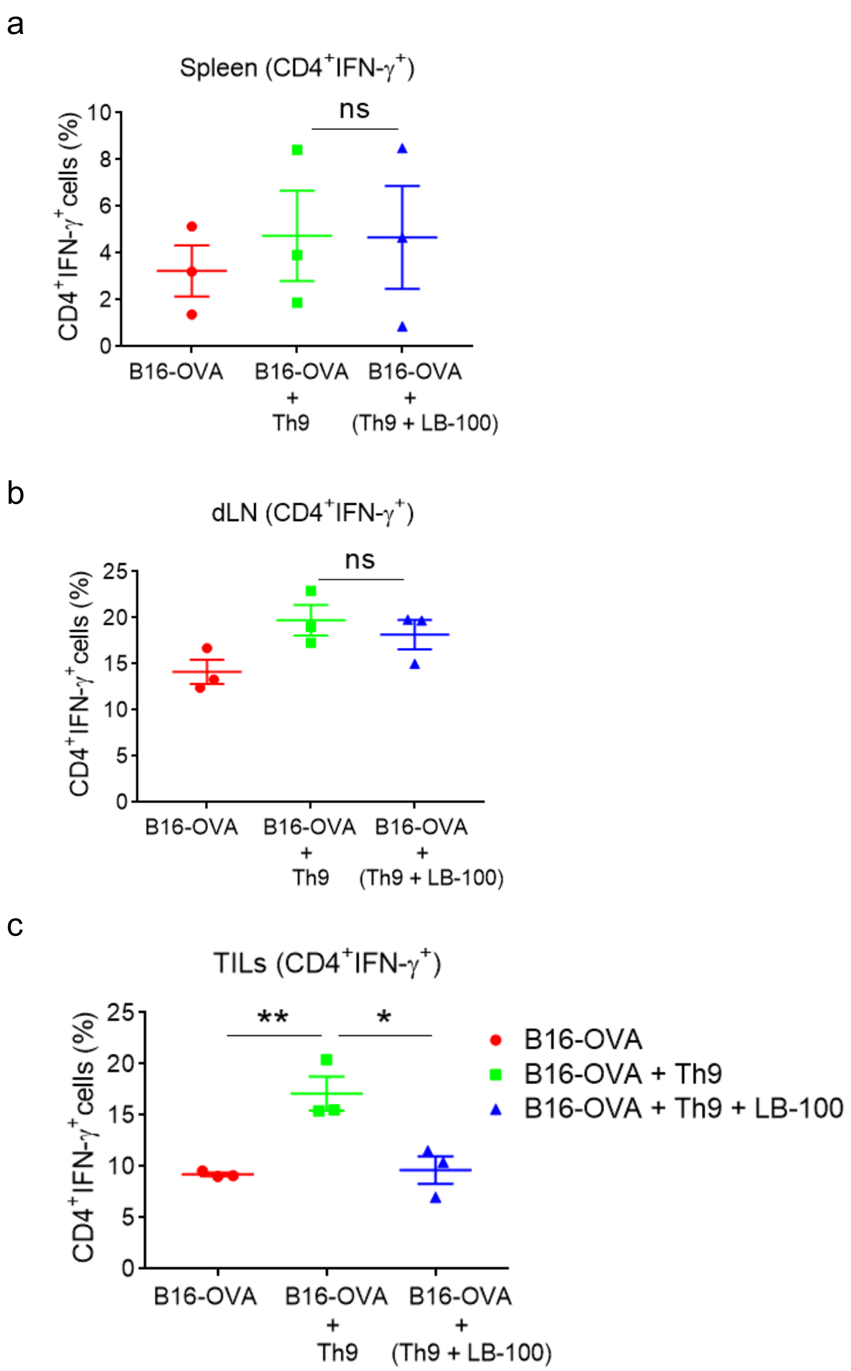

Supplementary Figure legends:

Supplementary Figure 1: Effect of IFN-γ neutralization on Th9 cells differentiation

Naïve $CD4^+$ T cells were treated with TGF-β1 (2.0 ng/ml) and IL-4 (20 ng/ml), in the absence or presence of increasing concentrations (0, 20, 50 μg/ml) of anti-IFN-γ respectively, followed by the flow cytometric analysis of intracellular IL-9 and IL-17 production.

Supplementary Figure 2: Protein-protein interaction in Th9 cells.

STRING based protein-protein interaction network of downregulated proteins in Th9 cells at >20 iBAQ intensity cut-off (red-phosphoproteins; blue-nucleotide-binding; green-ubiquitination).

Supplementary Figure 3: Effect of PP2A inhibitor on the proliferation and survival of Th9 cells

(a) Representative flow cytometry data for CFSE labelling demonstrating percentage of Th9 cells divided at different concentrations (0, 1, 2 ,5 μM) of LB-100. **(b)** Percentage of live Th9 cells was plotted against concentration of LB-100, and representative FACS plot showing the percentage of live Th9 cells with LB-100 dose titration. Data are representative of mean ± SEM from three independent experiments (n=3). ns - not significant; one-way ANOVA followed by Tukey's multiple comparison test **(b)**.

Supplementary Figure 4: PP2A inhibition suppressed IFN-γ expression in CD8+ T cells in tumor-bearing mice

TILs were isolated from the tumor while spleen and tumor draining lymph nodes (dLN) were harvested and single cell suspensions were made. Flow cytometric analysis of intracellular staining for $CD8^+$ IFN-γ^+ T cell populations in **(a)** spleen, **(b)** dLN and **(c)** TILs. Data represent one of three experiments with three independently analysed mice/group (n=3 mice per group).

Supplementary Figure 5: Effect of PP2A inhibition on IFN-γ expression in CD4+ T cells in tumor-bearing mice.

Spleen and tumor draining lymph nodes (dLN) were harvested and single cell suspensions were made. TILs were isolated from the tumor. Percentage of $CD4^+$ IFN-γ^+ T cell populations in **(a)** spleen, **(b)** dLN and **(c)** TILs. Data are representative of mean ± SEM from three independent experiments with three independently analysed mice/group (n=3 mice per group). *P<0.0332, **P<0.0021, ns - not significant; one-way ANOVA followed by Tukey's multiple comparison test **(a-c)**.

Supplementary Table S1: Total proteins in Th0 and Th9 on the basis of number of peptides.

Razor + Unique peptides	All peptides	>=2 peptides	>=3 peptides	>=4 peptides	>=5 peptides
Th0	2575	2150	1749	1416	1195
Th9	2566	2103	1709	1411	1163

Supplementary Table S2: 118 upregulated proteins in Th9 cells at log_{10} FC = +1

Protein ID	Gene Name	Protein Name
P45376	ALDR	Aldosereductase
Q8BRT1	CLAP2	CLIP-associating protein 2
P55937	GOGA3	Golgin subfamily A member 3
Q6P9L6	KIF15	Kinesin-like protein KIF15
Q5SV85	SYNRG	Synergingamma
P97789	XRN1	5'-3' exoribonuclease1
Q62077	PLCG1	1-phosphatidyl inositol 4,5 isphosphate phosphodiesterase gamma-1
Q00519	XDH	Xanthine dehydrogenase/oxidase
Q922J3	CLIP1	CAP-Glydomain-containing linker protein 1
Q58A65	JIP4	C-Jun-amino-terminal kinase-interacting protein 4
Q61543	GSLG1	Golgi apparatus protein 1
Q4QRL3	CC88B	Coiled-coil domain containing protein 88B
Q3UQ44	IQGA2	Ras GTPase-activating-like protein IQGAP 2
Q9QYE6	GOGA5	Golgin subfamily A member 5
O88379	BAZ1A	Bromo domain adjacent to zinc finger domain protein 1A
Q60520	SIN3A	Paired amphipathic helix protein Sin3a
Q9JJ28	FLII	Protein flightless-1 homolog
O08788	DCTN1	Dynactin subunit 1
Q8BX17	GEMI5	Gem-associated protein 5
Q8BY87	UBP47	Ubiquitin carboxyl-terminal hydrolase 47
Q9CXR1	DHRS7	Dehydrogenase/reductase SDR family member 7
Q9ES52	SHIP1	Phosphatidylinositol 3,4,5-trisphosphate 5-phosphatase 1
Q8CH18	CCAR1	Cell division cycle and apoptosis regulator protein 1
Q99PL5	RRBP1	Ribosome-binding protein 1
Q640N3	RHG30	Rho GTPase activating protein 30
Q3UPL0	SC31A	Protein transport protein Sec31A
Q7TSV4	PGM2	Phosphoglucomutase-2
Q8BL66	EEA1	Early endosome antigen 1
P33609	DPOLA	DNA polymerase alpha catalytic subunit
Q8BHG1	NRDC	Nardilysin
Q91VX2	UBAP2	Ubiquitin-associated protein 2
Q3UW53	NIBAN	Protein Niban 1
Q4VA53	PDS5B	Sister chromatid cohesion protein PDS5 homolog B
P38060	HMGCL	Hydroxymethylglutaryl-CoA lyase, mitochondrial
Q9JIY5	HTRA2	Serine protease HTRA2, mitochondrial
P59016	VP33B	Vacuolar protein sorting-associated protein 33B
Q3TBD2	HMHA1	Rho GTPase-activating protein 45
Q9JJ78	TOPK	Lymphokine-activated killer T-cell originated protein kinase
Q3UJB9	EDC4	Enhancer of mRNA decapping protein 4

P04627	ARAF	Serine/threonine-protein kinase A-Raf
Q9JKF7	RM39	39S ribosomal protein L39, mitochondrial
P07607	TYSY	Thymidylate synthase
Q64261	CDK6	Cyclin-dependent kinase 6
Q5EBH1	RASF5	Ras association domain containing protein 5
Q9D1J1	NECP2	Adaptin ear-binding coat-associated protein 2
Q3ULJ0	GPD1L	Glycerol-3-phosphate dehydrogenase1-like protein
Q64514	TPP2	Tripeptidyl-peptidase 2
Q9D8Y0	EFHD2	EF-hand domain-containing protein D2
Q8C129	LCAP	Leucyl-cystinyl aminopeptidase
O08808	DIAP1	Protein diaphanous homolog 1
Q91VA6	PDIP2	Polymerase delta interacting protein 2
Q9CY73	RM44	39S ribosomal protein L44, mitochondrial
Q9R1J0	NSDHL	Sterol-4-alpha-carboxylate 3-dehydrogenase, decarboxylating
Q9Z2D8	MBD3	Methyl-CpG-binding domain protein 3
Q80ZV0	RNH2B	Ribonuclease H2 subunit B
P62996	TRA2B	Transformer-2 protein homolog beta
Q9D1C9	RRP7A	Ribosomal RNA-processing protein 7 homolog A
Q91VE6	MK67I	MKI67 FHA domain-interacting nucleolar phosphoprotein
Q9CRD0	OCAD1	OCIA domain-containing protein 1
Q9DB96	NGDN	Neuroguidin
Q8R4R6	NUP53	Nucleoporin NUP53
O08915	AIP	AH receptor-interacting protein
P54923	ADPRH	[Protein ADP-ribosylarginine] hydrolase
Q06138	CAB39	Calcium-binding protein 39
O08579	EMD	Emerin
Q9CSU0	RPR1B	Regulation of nuclear pre-mRNA domain-containing protein 1B
Q9JII6	AK1A1	Alcohol dehydrogenase [NADP(+)]
Q9D1M0	SEC13	Protein SEC13 homolog
Q8BJU0	SGTA	Small glutamine-rich tetratricopeptide repeat-containing protein alpha
O70400	PDLI1	PDZ and LIM domain protein 1
Q9D1P4	CHRD1	Cysteine and histidine-rich domain-containing protein 1
Q9DAR7	DCPS	m7GpppX diphosphatase
Q3UGC7	EI3JA	Eukaryotic translation initiation factor 3 subunitJ-A
Q80X50	UBP2L	Ubiquitin-associated protein2-like
Q9D8B3	CHM4B	Charged multivesicular body protein 4b
O35864	CSN5	COP9 signalosome complex subunit 5
Q9JL26	FMNL1	Formin-like protein 1
Q9Z1Q5	CLIC1	Chloride intracellular channel protein 1
P54276	MSH6	DNA mismatch repair protein Msh6
P28352	APEX1	DNA-(apurinic or apyrimidinic site) lyase
E9Q7G0	NUMA1	Nuclear mitotic apparatus protein 1
P36552	HEM6	Oxygen-dependent coproporphyrinogen-III oxidase, mitochondrial

O70252	HMOX2	Heme oxygenase 2
P61965	WDR5	WD repeat-containing protein 5
Q9CVB6	ARPC2	Actin-related protein 2/3 complex subunit 2
Q9CW03	SMC3	Structural maintenance of chromosomes protein 3
O35855	BCAT2	Branched-chain-amino-acid aminotransferase, mitochondrial
P51863	VA0D1	V-type proton ATPase subunit d1
Q6P5E4	UGGG1	UDP-glucose:glycoprotein glucosyltransferase 1
P63330	PP2AA	Serine/threonine-protein phosphatase2A catalytic subunit alpha isoform
E9Q557	DESP	Desmoplakin
Q8VHZ7	IMP4	U3 small nucleolar ribonucleoprotein protein IMP4
Q9CYG7	TOM34	Mitochondrial import receptor subunit TOM34
Q6ZWX6	IF2A	Eukaryotic translation initiation factor 2 subunit 1
P10711	TCEA1	Transcription elongation factor Aprotein 1
Q9QZD9	EIF3I	Eukaryotic translation initiation factor 3 subunit
O89100	GRAP2	GRB2-related adaptor protein 2
P14483	HB2A	H-2 class II histocompatibility antigen, A beta chain
Q9Z277	BAZ1B	Tyrosine-protein kinase BAZ1B
Q6ZQ58	LARP1	La-related protein 1
Q99P88	NUP155	Nuclearpore complex protein Nup155
P24063	ITAL	Integrin alpha-L
P52825	CPT2	Carnitine O-palmitoyltransferase 2, mitochondrial
Q9JJF3	RIOX1	Ribosomal oxygenase 1
O88545	CSN6	COP9 signalosome complex subunit 6
Q62393	TPD52	Tumor protein D52
Q02257	PLAK	Junction plakoglobin
Q9DC48	PRP17	Pre-mRNA-processing factor 17
Q9CT10	RANB3	Ran-binding protein 3
Q3TBT3	STING	Stimulator of interferon genes protein
P23492	PNPH	Purine nucleoside phosphorylase
P51125	ICAL	Calpastatin
Q9CZ04	CSN7A	COP9 signalosome complex subunit 7a
P35235	PTN11	Tyrosine-protein phosphatase non-receptor type 11
Q9CU62	SMC1A	Structural maintenance of chromosomes protein 1A
Q9JKR6	HYOU1	Hypoxia up-regulated protein 1
O08734	BAK	Bcl-2 homologous antagonist/killer
Q99J62	RFC4	Replication factor C subunit 4

Supplementary Table S3: 81 downregulated proteins in Th9 cells at log_{10} FC = -1

Protein ID	Gene Name	Protein Name
P58044	IDI1	Isopentenyl-diphosphate Delta-isomerase 1
Q3U1J4	DDB1	DNA damage-binding protein 1
Q6PAM1	TXLNA	Alpha-taxilin
Q8JZK9	HMCS1	Hydroxymethylglutaryl-CoA synthase, cytoplasmic
Q9DCA5	BRX1	Ribosome biogenesis protein BRX1 homolog
Q8C3I8	HGH1	Protein HGH1 homolog
Q9EQP2	EHD4	EH domain-containing protein 4
Q922V4	PLRG1	Pleiotropic regulator 1
Q9ERI2	RB27A	Ras-related protein Rab-27A
Q8BFP9	PDK1	[Pyruvate dehydrogenase(acetyl-transferring)] kinase isozyme1, mitochondrial
Q8K4Z5	SF3A1	Splicing factor 3A subunit 1
Q8VEH6	CBWD1	COBW domain-containing protein 1
O08756	HCD2	3-hydroxyacyl-CoA dehydrogenase type-2
O08807	PRDX4	Peroxiredoxin-4
P37913	DNLI1	DNA ligase 1
Q9CXF4	TBC15	TBC1 domain family member 15
Q8R5H1	UBP15	Ubiquitin carboxyl-terminal hydrolase 15
P70697	DCUP	Uroporphyrinogen decarboxylase
Q8K310	MATR3	Matrin-3
Q9QXB9	DRG2	Developmentally-regulated GTP-binding protein 2
Q9Z2U0	PSA7	Proteasome subunit alpha type-7
P47738	ALDH2	Aldehyde dehydrogenase, mitochondrial
O08709	PRDX6	Peroxiredoxin-6
Q8R2M2	TDIF2	Deoxynucleotidyl transferase terminal-interacting protein
Q9EPU0	RENT1	Regulator of nonsense transcripts 1
Q8K2M0	RM38	39S ribosomal protein L38, mitochondrial
Q99MR6	SRRT	Serrate RNA effector molecule homolog
Q6PB66	LPPRC	Leucine-rich PPR motif-containing protein, mitochondrial
P17751	TPIS	Triose phosphate isomerase
P27048	RSMB	Small nuclear ribonucleoprotein-associated protein B
P62827	RAN	GTP-binding nuclear protein Ran
Q5SWU9	ACACA	Acetyl-CoA carboxylase 1
Q3UQ84	SYTM	Threonine—tRNA ligase, mitochondrial
Q5DTM8	BRE1A	E3 ubiquitin-protein ligase BRE1A
Q9Z1T1	AP3B1	AP-3 complex subunit beta-1
Q6P9R1	DDX51	ATP-dependent RNA helicase DDX51
Q91ZA3	PCCA	Propionyl-CoA carboxylase alpha chain, mitochondrial
P58501	PAXB1	PAX3-and PAX7-binding protein 1
P70268	PKN1	Serine/threonine-protein kinase N1
Q9JIG7	CCD22	Coiled-coil domain-containing protein 22

Q8BZQ7	ANC2	Anaphase-promoting complex subunit 2
Q924C1	XPO5	Exportin-5
Q8BRN9	C2D1B	Coiled-coil and C2 domain-containing protein 1B
Q8C7R4	UBA6	Ubiquitin-like modifier-activating enzyme 6
Q8CFI7	RPB2	DNA-directed RNA polymerase II subunit RPB2
Q64727	VINC	Vinculin
O70475	UGDH	UDP-glucose 6-dehydrogenase
Q8BH86	GLUCM	D-glutamate cyclase, mitochondrial
P09055	ITB1	Integrin beta-1
Q9WU62	INCE	Inner centromere protein
Q8JZM7	CDC73	Parafibromin
Q9JLI8	SART3	Squamous cell carcinoma antigen recognized by T-cells 3
Q8K4F6	NSUN5	Probable 28S rRNA (cytosine-C(5))-methyltransferase
Q8R5K4	NOL6	Nucleolar protein 6
Q8VDP3	MICA1	[F-actin]-methionine sulfoxide oxidase MICAL1
Q99MN9	PCCB	Propionyl-CoA carboxylase beta chain, mitochondrial
P52431	DPOD1	DNA polymerase delta catalytic subunit
P55302	AMRP	Alpha-2-macroglobulin receptor-associated protein
Q8BIW1	PRUN1	Exopolyphosphatase PRUNE 1
Q9Z2X8	KEAP1	Kelch-like ECH-associated protein 1
Q8VDP4	CCAR2	Cell cycle and apoptosis regulator protein 2
B9EJ86	OSBL8	Oxysterol-binding protein-related protein 8
Q9JHK4	PGTA	Geranylgeranyl transferase type-2 subunit alpha
P27046	MA2A1	Alpha-mannosidase 2
Q3UHD6	SNX27	Sortingnexin-27
Q9JI13	SAS10	Something about silencing protein 10
P52432	RPAC1	DNA-directed RNA polymerases I and III subunit RPAC1
Q9DC71	RT15	28S ribosomal protein S15, mitochondrial
Q91W39	NCOA5	Nuclear receptor coactivator 5
Q6GQT9	NOMO1	Nodal modulator 1
Q9CY27	TECR	Very-long-chain enoyl-CoA reductase
P68181	KAPCB	cAMP-dependent protein kinase catalytic subunit beta
Q810A7	DDX42	ATP-dependent RNA helicase DDX42
Q9CX99	GRAP	GRB2-related adapter protein
Q9DBL7	COASY	Bifunctional coenzyme A synthase
Q99LP6	GRPE1	GrpE protein homolog 1,mitochondrial
Q6P9P6	KIF11	Kinesin-like protein KIF11
Q8K224	NAT10	RNA cytidine acetyltransferase
P70404	IDHG1	Isocitrate dehydrogenase [NAD] subunit gamma 1, mitochondrial
Q64105	SPRE	Sepiapterin reductase
Q62422	OSTF1	Osteoclast-stimulating factor 1

Supplementary Table S4: STRING based pathway analysis of upregulated proteins in Th9 cells at log_{10} FC= +1

Pathway ID	Pathway Name	Gene symbol
MMU-168256	Immune System	Arpc2, Atp6v0d1, Cab39, Dctn1, Diap1, Dsp, Eea1, Grap2, H2Ab1, Hmha1, Hmox2, Inpp5d, Iqgap2, Itgal, Jup, Kif15, Lnpep, Pgm1, Plcg1, **Ppp2ca**, Ptpn11, Sec13, Sec31a, Tmem173, Tpp2, Xdh
MMU-69278	Cell Cycle, Mitotic	Cdk6, Clip1, Dctn1, Emd, Numa1, Nup155, Pds5b, Pola1, **Ppp2ca**, Rfc4, Sec13, Smc1a, Smc3
MMU-168249	Innate Immune System	Arpc2, Atp6v0d1, Cab39, Diap1, Dsp, Eea1, Grap2, Hmha1, Hmox2, Iqgap2, Itgal, Jup, Pgm1, Plcg1, **Ppp2ca**, Ptpn11, Tmem173
MMU-68877	Mitotic Prometaphase	Clip1, Dctn1, Numa1, Pds5b, **Ppp2ca**, Sec13, Smc1a, Smc3
MMU-68886	M Phase	Clip1, Dctn1, Emd, Numa1, Nup155, Pds5b, **Ppp2ca**, Sec13, Smc1a, Smc3
MMU-1280218	Adaptive Immune System	Dctn1, Grap2, H2Ab1, Inpp5d, Itgal, Kif15, Lnpep, Plcg1, **Ppp2ca**, Ptpn11, Sec13, Sec31a, Tpp2, Xdh
MMU-199991	Membrane Trafficking	Arpc2, Chmp4b, Cops5, Cops6, Cops7a, Dctn1, Golga5, Kif15, Necap2, Sec13, Sec31a, Tpd52
MMU-2468052	Establishment of Sister Chromatid Cohesion	Pds5b, Smc1a, Smc3
MMU-2470946	Cohesin Loading onto Chromatin	Pds5b, Smc1a, Smc3
MMU-2500257	Resolution of Sister Chromatid Cohesion	Clip1, Pds5b, **Ppp2ca**, Sec13, Smc1a, Smc3
MMU-6781827	Transcription-Coupled Nucleotide Excision Repair (TC-NER)	Cops5, Cops6, Cops7a, Rfc4, Tcea1
MMU-210990	PECAM1 interactions	Inpp5d, Plcg1, Ptpn11
MMU-73894	DNA Repair	Apex1, Baz1b, Cops5, Cops6, Cops7a, Msh6, Rfc4, Tcea1
MMU-6781823	Formation of TC-NER Pre-Incision Complex	Cops5, Cops6, Cops7a, Tcea1
MMU-202433	Generation of second messenger molecules	Grap2, H2-Ab1, Plcg1
MMU-202733	Cell surface interactions at the vascular wall	Glg1, Inpp5d, Itgal, Plcg1, Ptpn11

MMU-388841	Costimulation by the CD28 family	Grap2 ,H2-Ab1, **Ppp2ca**, Ptpn11
MMU-6798695	Neutrophil degranulation	Cab39, Diap1, Dsp, Hmha1, Hmox2, Iqgap2, Itgal, Jup, Pgm1, Tmem173
MMU-195258	RHO GTPase Effectors	Arpc2, Clip1, Diap1, Fmnl1, Iqgap2, **Ppp2ca**, Sec13
MMU-2467813	Separation of Sister Chromatids	Clip1, Pds5b, **Ppp2ca**, Sec13, Smc1a, Smc3
MMU-2132295	MHC class II antigen presentation	Dctn1, H2-Ab1, Kif15, Sec13, Sec31a
MMU-8856828	Clathrin-mediated endocytosis	Arpc2, Cops5, Cops6, Cops7a, Necap2
MMU-194315	Signaling by Rho GTPases	Arpc2, Clip1, Diap1, Fmnl1, Hmha1, Iqgap2, **Ppp2ca**, Sec13
MMU-5663220	RHO GTPases Activate Formins	Clip1, Diap1, Fmnl1, **Ppp2ca**, Sec13
MMU-5696399	Global Genome Nucleotide Excision Repair (GG-NER)	Cops5, Cops6, Cops7a, Rfc4
MMU-8953854	Metabolism of RNA	Ccar1, Cdc40, Dcps, Gemin5, Imp4, Nup155, **Ppp2ca**, Rrp7a, Tra2b
MMU-5696394	DNA Damage Recognition in GG-NER	Cops5, Cops6, Cops7a
MMU-69242	S Phase	Pds5b, Pola1, Rfc4, Smc1a, Smc3
MMU-8934593	Regulation of RUNX1 Expression and Activity	Cdk6, Ptpn11
MMU-392499	Metabolism of proteins	Cops5, Cops6, Cops7a, Dctn1, Eif2s1, Eif3i, Eif3j1, Mrpl39, Mrpl44, Nup155, Sec13, Sec31a, Sin3a, Smc1a, Smc3, Uggt1, Usp47, Wdr5
MMU-8856825	Cargo recognition for clathrin-mediated Endocytosis	Cops5, Cops6, Cops7a, Necap2
MMU-72766	Translation	Eif2s1, Eif3i, Eif3j1, Mrpl39, Mrpl44
MMU-113501	Inhibition of replication initiation of damaged DNA by RB1/E2F1	Pola1, **Ppp2ca**
MMU-5654743	Signaling by FGFR4	Plcg1, **Ppp2ca**, Ptpn11
MMU-72695	Formation of the ternary complex, and subsequently, the 43S complex	Eif2s1, Eif3i, Eif3j1

MMU-5654741	Signaling by FGFR3	Plcg1, **Ppp2ca**, Ptpn11
MMU-202403	TCR signaling	Grap2, H2-Ab1, Inpp5d, Plcg1
MMU-5654736	Signaling by FGFR1	Plcg1, **Ppp2ca**, Ptpn11
MMU-72702	Ribosomal scanning and start codon Recognition	Eif2s1, Eif3i, Eif3j1
MMU-74160	Gene expression (Transcription)	Baz1b, Cdc40, Cdk6, Mbd3, Nup155, **Ppp2ca**, Ptpn11, Rfc4, Rprd1b, Sin3a, Tcea1, Wdr5
MMU-1295596	Spry regulation of FGF signaling	**Ppp2ca**, Ptpn11
MMU-72649	Translation initiation complex formation	Eif2s1, Eif3i, Eif3j1
MMU-156827	L13a-mediated translational silencing of Ceruloplasmin expression	Eif2s1, Eif3i, Eif3j1
MMU-174411	Polymerase switching on the C-strand of the Telomere	Pola1, Rfc4
MMU-69091	Polymerase switching	Pola1,Rfc4
MMU-9006934	Signaling by Receptor Tyrosine Kinases	Atp6v0d1, Diap1, Grap2, Jup, Plcg1, **Ppp2ca**, Ptpn11
MMU-389948	PD-1 signaling	H2-Ab1, Ptpn11
MMU-5651801	PCNA-Dependent Long Patch Base Excision Repair	Apex1, Rfc4
MMU-189445	Metabolism of porphyrins	Cpox, Hmox2
MMU-5654738	Signaling by FGFR2	Plcg1, **Ppp2ca**, Ptpn11
MMU-3108214	SUMOylation of DNA damage response and repair proteins	Nup155, Smc1a, Smc3
MMU-389513	CTLA4 inhibitory signaling	**Ppp2ca**, Ptpn11
MMU-3108232	SUMO E3 ligases SUMOylate target proteins	Nup155, Sin3a, Smc1a, Smc3
MMU-432142	Platelet sensitization by LDL	**Ppp2ca**,Ptpn11
MMU-186763	Downstream signal transduction	Plcg1, Ptpn11

MMU-2871809	FCERI mediated Ca+2 mobilization	Grap2, Plcg1
MMU-5673000	RAF activation	Araf, **Ppp2ca**
MMU-73857	RNA Polymerase II Transcription	Cdc40, Cdk6, Mbd3, **Ppp2ca**, Ptpn11, Rfc4, Rprd1b, Sin3a, Tcea1, Wdr5

Supplementary Table S5: STRING based pathway analysis of downregulated proteins in Th9 cells at $\log_{10}$ FC= -1

Pathway ID	Pathway Name	Gene symbol
MMU-196780	Biotin transport and metabolism	Acaca,Pcca,Pccb
MMU-8953854	Metabolism of RNA	Ddx42,Nol6,Plrg1,Polr2b,Psma7,Sf3a1,Snrpb,Srrt,Upf1,Utp3
MMU-72163	mRNA Splicing - Major Pathway	Ddx42,Plrg1,Polr2b,Sf3a1,Snrpb,Srrt
MMU-71032	Propionyl-CoA catabolism	Pcca,Pccb
MMU-196849	Metabolism of water-soluble vitamins and cofactors	Acaca,Coasy,Pcca,Pccb
MMU-72165	mRNA Splicing - Minor Pathway	Ddx42,Polr2b,Snrpb
MMU-392499	Metabolism of proteins	Ccdc22,Cdc73,Ddb1,Incenp,Keap1,Man2a1,Mrpl38,Mrps15, Psma7,Rab27a,Rabggta,Rnf20,Uba6,Usp15
MMU-6782135	Dual incision in TC-NER	Ddb1,Pold1,Polr2b
MMU-6782210	Gap-filling DNA repair synthesis and ligation in TC-NER	Ddb1,Pold1,Polr2b
MMU-8852135	Protein ubiquitination	Cdc73,Rnf20,Uba6
MMU-597592	Post-translational protein modification	Ccdc22,Cdc73,Ddb1,Incenp,Keap1,Man2a1,Psma7,Rab27a, Rabggta,Rnf20,Uba6,Usp15
